# Juvenile Rectal Polyp Exhibiting Osseous Metaplasia: A Case Report

**DOI:** 10.1155/crpe/8495794

**Published:** 2025-10-28

**Authors:** Antonio Marseglia, Fabio Rotondo, Anna Locatelli, Paola Parente, Dalila Tedeschi, Maria Rosa Pastore, Massimo Pettoello Mantovani

**Affiliations:** ^1^Department of Pediatrics, Fondazione IRCCS “Casa Sollievo Della Sofferenza”, San Giovanni Rotondo, Italy; ^2^Department of Pediatrics, University of Foggia, Foggia, Italy; ^3^Department of Pathology, Fondazione IRCCS “Casa Sollievo Della Sofferenza”, San Giovanni Rotondo, Italy

## Abstract

A 10-year-old boy presented with painless rectal bleeding. No fever or weight loss was reported. He had no history of constipation. Ileocolonoscopy revealed a single 2 cm pedunculated polyp in the distal rectum, which was removed endoscopically. Histological examination demonstrated polypoid tissue with focal ulceration characterized by an inflamed lamina propria containing dilated, branched, and hyperplastic crypts. Higher-power microscopy revealed mature bony trabeculae within the lamina propria. No dysplasia was observed. Osseous metaplasia is generally regarded as clinically and prognostically insignificant and is typically an incidentalhistological finding. We report an extremely rare case of heterotopic bone formation in a juvenile rectal polyp and review recent literature on this phenomenon.

## 1. Background

Gastrointestinal polyps are frequently identified in early childhood and represent one of the most common causes of painless rectal bleeding in children [1]. Most polyps are solitary, confined to the colon, and pose no long-term neoplastic risk. Endoscopically, they may appear flat (sessile) or pedunculated. Histologically, polyps are classified into two major categories: hamartomas and adenomas.

Hamartomatous polyps (so called “juvenile inflammatory polyps”) are usually pedunculated lesions covered by friable, granular tissue that bleeds easily and often show a dense inflammatory infiltrate, leading to ulceration, cystic dilatation of glands, and the formation of granulation tissue within the lamina propria. Hamartomas are nonneoplastic and only rarely exhibit dysplastic changes.

Adenomatous polyps are dysplastic and considered precancerous lesions. In pediatric patients, solitary polyps are most often hamartomatous, while adenomatous polyps are extremely rare. Adenomas are typically seen in familial polyposis syndromes [2]; therefore, the detection of multiple polyps should prompt evaluation for syndromes such as juvenile polyposis syndrome (JPS), Peutz–Jeghers syndrome (PJS), familial adenomatous polyposis (FAP), and MUTYH-associated polyposis (MAP) [2, 3].

Heterotopic bone formation (osseous metaplasia) in gastrointestinal polyps has been rarely reported [4], although most reported cases involve adenomas or malignant lesions, scattered case reports and small series have documented osseous metaplasia in benign juvenile polyps over the past decade [4–7]. In this report, we describe a rare case of osseous metaplasia in a juvenile hamartomatous rectal polyp in a 10-year-old male and review the literature regarding its etiology and pathogenesis.

## 2. Case Report

A 10-year-old male was referred to our Pediatric Gastroenterology Unit for a 4-month history of painless rectal bleeding. Episodes were occasionally preceded by mild lower abdominal discomfort, but did not significantly interfere with his daily activities. He denied fever, weight loss, or constipation. Family history was significant for inflammatory bowel disease. On admission, physical examination was unremarkable. The abdomen was flat,nontender, and nondistended, except for slight discomfort in the lower quadrants. No visceromegaly was detected. Anal inspection revealed no fissures or fistulas; the anal orifice and mucosa appeared normal. Digital rectal examination was not performed.

Laboratory blood tests showed mild iron deficiency (hemoglobin 13.7 g/dL, serum iron 49 μg/dL, ferritin 20 ng/mL, and transferrin 326 mg/dL). Coagulation profile, inflammatory markers, and fecal calprotectin were within normal limits. Infectious disease screening was negative.

Colonoscopy revealed a single pedunculated polyp measuring approximately 2 cm in diameter in the distal rectum; the remainder of the colon was normal. Multiple biopsies of the macroscopically normal ascending and transverse colon showed preserved crypt architecture and no inflammatory infiltrate or granulomas. A polypectomy was performed under general anesthesia, and the specimen was submitted for histopathological examination.

**Figure 1:**
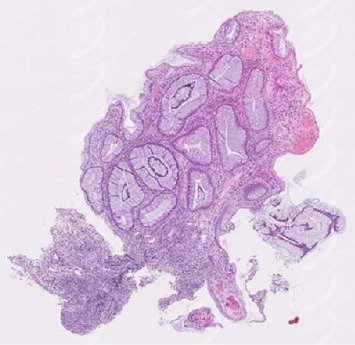
Low-power view showing cystically dilated glands with inflamed, edematous stroma, typical of a juvenile rectal polyp.

**Figure 2:**
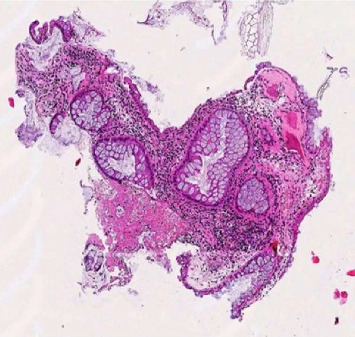
Intermediate magnification highlighting glandular distortion, prominent chronic inflammatory infiltrate, and early stromal mineralization.

**Figure 3:**
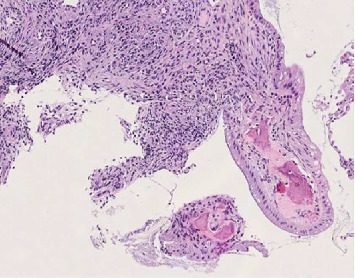
High-power view demonstrating mature lamellar bone trabeculae within the polyp stroma, consistent with osseous metaplasia.

Histologic evaluation with hematoxylin and eosin staining revealed polypoid tissue with focal ulceration, composed of inflamed lamina propria and dilated, branched, and hyperplastic crypts (Figure 1). Higher-power microscopy demonstrated mature bony trabeculae within the lamina propria (Figures 2 and 3). No dysplasia was identified. Adjacent nonpolypoid mucosa at the polypectomy margin displayed normal colonic histology without microscopic inflammation. Histological examination of biopsy specimens taken from the ascending and transverse colon showed preserved crypt architecture without evidence of chronic inflammatory infiltrate, granulomas, or basal plasmacytosis.

## 3. Discussion

Osseous metaplasia in gastrointestinal polyps is exceedingly rare and represents a poorly understood histopathological finding. Most reported cases have occurred in adenomatous or malignant lesions [8, 9], with fewer than 20 well-documented cases in juvenile polyps reported since 2010 [4–6, 10–12]. The rectum and left colon are most frequently involved, likely reflecting the higher prevalence of inflammatory and neoplastic lesions in these regions, which provide a favorable environment for stromal osteogenic transformation. In contrast, cases involving the ileum, stomach, and Barrett's esophagus are exceptional [7].

The exact mechanism of heterotopic bone formation in colonic polyps remains unclear [5]. Several hypotheses have been proposed. In 1964, Marks and Atkinson suggested that osseous metaplasia results from the transformation of fibroblasts into osteoblasts [8]. In 1989, Randall et al. proposed that alkaline phosphatase expression, a marker of osteoblast differentiation, by proliferating mesenchymal cells may play a role. More recently, many authors have implicated bone morphogenetic proteins (BMPs) in the pathogenesis of osseous metaplasia [5, 13]. BMPs, members of the transforming growth factor-β (TGF-β) superfamily, may promote local expression of substance P following tissue injury via paracrine mechanisms. Substance P stimulates local inflammation, which can lead to heterotopic ossification. Kypson et al. demonstrated BMP2 expression in rectal adenocarcinoma with osseous metaplasia [9].

Furthermore, in vitro studies have shown that human fibroblast cultures exposed to specific transcription factors (Oct3/4, Sox2, c-Myc, and Klf4) can be reprogrammed into pluripotent stem cells capable of differentiating into various cell types, including osteoblasts. It is hypothesized that mature fibroblasts within the stromal component of polyps may undergo similar reprogramming under the influence of these factors, resulting in osteoblastic differentiation [5, 14, 15]. More recent contributions to literature have enhanced our understanding of the potential mechanisms underlying osseous metaplasia in this setting. Nguyen et al. reported elevated interleukin-6 (IL-6) expression in juvenile rectal polyps exhibiting focal ossification, implicating proinflammatory cytokines in the pathogenesis of osteogenic transdifferentiation [16]. A more recent multicenter review by Santos et al. identified overexpression of microRNA-21 (miR-21) in nearly 50% of similar cases. microRNA-21 promotes SMAD signaling and downregulates known inhibitors of osteoblastogenesis, such as PTEN and SPRY2, amplifying osteoinductive pathways [17].

Clinically, the presence of osseous metaplasia within a juvenile polyp is of uncertain significance. It is typically an incidental histological finding without prognostic implication. However, its identification is crucial to avoid diagnostic confusion with dystrophic calcifications, ossifying neoplasms, or metastatic bone deposits. From a scientific standpoint, these rare cases offer a unique window into the processes of mesenchymal differentiation and tissue remodeling in the gastrointestinal tract.

This report contributes to the limited but expanding literature on heterotopic ossification in juvenile colorectal polyps. Nevertheless, the precise etiology and molecular mediators of heterotopic ossification in juvenile polyps remain incompletely understood. Further studies, particularly those employing molecular profiling and lineage tracing, are needed to elucidate the precise cellular origins and regulatory networks involved in this rare phenomenon [18].

## 4. Conclusion

Osseous metaplasia is an incidental, clinically insignificant finding in colonic polyps. We report a rare case of heterotopic bone formation within a juvenile rectal polyp, highlighting its distinctive histological features.

## Data Availability

The data that support the findings of this study are available from the corresponding author upon reasonable request.

## Nomenclature

JPS: Juvenile polyposis syndrome
PJS: Peutz–Jeghers syndrome
FAP: Familial adenomatous polyposis
AFAP: Attenuated familial adenomatous polyposis
MAP: MUTYH-associated polyposis (MAP)
BMPs: Bone morphogenetic proteins
